# News Fluorescence-Based Polarization Immunoassays as a Frontline Test for the Rapid Detection of Acute and Late Phase Lyme-Borreliosis Disease

**DOI:** 10.3390/molecules31183152

**Published:** 2026-09-08

**Authors:** Joao P. R. S. Carvalho, Monica E. T. Alcón-Chino, Paloma Napoleão-Pêgo, Guilherme C. Lechuga, Isis C. Prado, Mariana S. Freitas, Jessica A. Waterman, Karyne Rangel, Salvatore G. De-Simone

**Affiliations:** 1 Center for Technological Development in Health (CDTS)/National Institute of Science and Technology for Innovation in Neglected Population Diseases (INCT-IDPN), Oswaldo Cruz Foundation (FIOCRUZ), Rio de Janeiro 21040-900, Brazil; joaopedrorsc@gmail.com (J.P.R.S.C.); pegopn@gmail.com (P.N.-P.); gclechuga@gmail.com (G.C.L.); isis.prado@ioc.fiocruz.br (I.C.P.); freitasmarii97@gmail.com (M.S.F.); jessicawaterman@aluno.fiocruz.br (J.A.W.); karyne.rangelk@gmail.com (K.R.); 2 Program of Post-Graduation on Science and Biotechnology, Department of Molecular and Cellular Biology, Biology Institute, Federal Fluminense University, Niteroi 22040-036, Brazil; elizaalcon@gmail.com; 3 Program of Post-Graduation on Parasitic Biology, Oswaldo Cruz Institute, Oswaldo Cruz Foundation, Rio de Janeiro 21040-900, Brazil; 4 Epidemiology and Molecular Systematics Laboratory (LEMS), Oswaldo Cruz Institute, FIOCRUZ, Rio de Janeiro 21040-900, Brazil

**Keywords:** borreliosis, IgM and IgG epitopes, fluorescence polarization immunoassay, rapid testing method, acute and late diagnostic

## Abstract

Lyme borreliosis (LB) is a tick-borne disease caused by a diverse and expanding group of spirochetes characterized by complex biology and advanced immune evasion mechanisms. It presents a wide range of clinical symptoms affecting multiple organ systems and can lead to persistent complications. The pathogenesis of LB remains incompletely understood, and diagnosis typically relies on serologic assays to detect antibodies against LB. However, the standard two-tiered testing (STTT) algorithm is limited by cross-reactivity, low sensitivity, and delayed results, hindering timely and accurate diagnosis. Although molecular tests are considered the gold standard, their reliance on centralized laboratories can delay critical treatment decisions. This underscores the urgent need for rapid, reliable diagnostic tools, particularly for use at the point of hospital admission. Point-of-care serological and direct antigen testing can provide actionable information, supporting decentralized healthcare systems in diagnosing complex diseases, such as LB. In this study, we developed two fluorescent polarization immunoassays (FPIAs) using IgM and IgG LB-specific synthetic epitopes/peptides to evaluate their diagnostic potential. These FPIAs showed high sensitivity and specificity in detecting IgM or IgG anti-LB antibodies in patient sera within minutes. The fluorescently labeled synthetic peptides produced significant polarization differences between infected and healthy samples, allowing clear discrimination. The FPIA-LB functions as a one-step assay, eliminating the need for secondary antibodies or complex protocols. This work highlights the FPIA technique as a robust, rapid, and efficient tool for LB diagnosis, offering a promising advance in improving early detection and patient outcomes, which could save lives and reduce long-term health complications.

## 1. Introduction

Numerous bacteria belonging to the sensu lato complex of *Borrelia* are responsible for Lyme-Borreliosis (LB), an increasingly common disease. Recently, studies have identified various *Borrelia* species in different countries and even within the same region, exhibiting distinct genomes (genospecies) [1,2,3,4,5]. These findings are supported by surveys in endemic areas, such as Siberia, where a wide spectrum of *Ixodidae* ticks harbors diverse tick-borne bacterial pathogens, including *Borrelia* spp. [6]. These genospecies exhibit variations in clinical presentation, dissemination within the human body, and expression of genomes and antigens [7]. This variation in antigenic expression across geographic regions and even within localized areas creates significant challenges in diagnosing and managing the disease, particularly in its advanced stages.

Lyme-Borreliosis is a multi-systemic disease that is preventable and treatable if identified early. Raising awareness, ensuring timely diagnosis, and providing proper treatment are essential for reducing its burden and avoiding long-term complications. Early-stage clinical diagnosis of LB is critical, as the disease can be mistaken for other infectious or autoimmune disorders. Misdiagnosis can result in inappropriate treatment, leading to prolonged infection and a strong antibody response that fails to eliminate the pathogen.

The most widely used laboratory approach for diagnosing LB is the standard two-tier serology (STT), which involves an initial enzyme-linked immunosorbent assay (ELISA or EIA) to detect antibodies against *Borrelia burgdorferi*, followed, if results are positive or equivocal, by a confirmatory Western blot targeting specific *Borrelia* proteins [8,9]. A more recent variation, the modified two-tiered testing (MTTT) algorithm, replaces the Western Blot with a second ELISA, offering improved sensitivity, especially in early-stage Lyme disease, and was recently approved for use in the United States [10,11,12]. While widely used, this method has limitations, such as false-negative results in the early stages (before antibodies are produced) and false positives due to cross-reactions with other infections [13]. PCR offers an alternative with high specificity, though its sensitivity depends on the sample type and disease stage [14,15,16]. Several variants have raised severe public health concerns, as they may have increased the potential for human-to-human transmission. In molecular diagnostic laboratories, RT-PCR has been the gold standard for detecting emerging. Advancing diagnostic platforms using single or multiple subunit antigens that target unique *Borrelia* epitopes could improve specificity and sensitivity, thereby reducing false positives in human borreliosis [17,18,19].

*Borrelia* spreads from the tick bite site as the disease progresses, causing early disseminated symptoms such as multiple erythema migrans, carditis, and meningitis. If left untreated, LB may progress to a late stage, resulting in severe complications like arthritis and encephalitis. In rare cases, skin manifestations such as *acrodermatitis chronica atrophicans* may occur, particularly in patients with underlying conditions, such as sarcoidosis [20]. While early antibiotic treatment is highly effective, delays in diagnosis or therapy make the disease more challenging to manage and lead to significant health consequences [21].

Molecular biology tests are alternative assays that are more sensitive, but they are typically carried out at central laboratories, which can delay treatment and control decisions. They have several general drawbacks, including high costs, dependence on expensive equipment, and the inability to obtain quick results on-site. These tests typically involve multistep operations, which can lead to increased cumulative errors in detection.

Due to their great potential for on-site applications, such as environmental testing and point-of-care diagnostics (POCT), single-step immunoassays have attracted considerable attention for the quantification of target analytes [22,23,24]. The quick outcomes and simplicity of use of these techniques make them popular. One popular type of homogeneous single-step immunoassay is the fluorescence polarization immunoassay (FPIA). FPIAs do not require laborious procedures such as bound-free separation or repeated washing, unlike other techniques used in the food analysis, clinical, and biomedical domains [25,26,27,28]. Different formats of FPIAs have been evaluated for various infectious diseases [29,30] in both animals and humans. For instance, FPIA has proven useful for detecting antibodies against bacterial contamination and in viral diagnostics. Beyond diagnostics, this technology has also been used in high-throughput screening to identify protein-protein interaction inhibitors in parasitic diseases such as leishmaniasis, demonstrating its broad applicability [31]. In animals, FPIA has been proven useful for detecting antibodies against bacterial contamination, particularly in detecting antibodies to gram-negative bacteria [32], equine infectious anemia virus [33], *Mycobacterium bovis* [34,35], brucellosis [36,37,38], H5 avian influenza virus [39], and influenza A virus in chicken and goat sera [40]. In humans, it has been used for immunoassays [41] and in the detection of COVID-19 [42,43].

The FPIA is based on the idea that randomly rotating molecules in a solution causes plane-polarized light to become depolarized. Fluorescein-bound antigens that react with antibodies exhibit less movement, resulting in less light depolarization, as this movement is inversely proportional to the molecular weight. This principle has been successfully applied to detect protein–ligand interactions with high sensitivity, such as in the development of lipopolysaccharide-caspase-4 interaction assays [44]. The FPIA can measure this change in the rate of depolarization in milliPolarization units, offering quick and reliable test results [45,46].

Conversely, synthetic peptides have been gaining prominence in diagnostic tests due to their low cost, rapid production (eliminating the need for cell culture and protein purification), and high sensitivity and specificity [47,48]. Moreover, fluorescence polarization assays require small molecules, such as peptides, to effectively measure interactions between target biomolecules. Thus, selecting low-molecular-weight tracers is crucial when designing the assay [30,49,50,51]. Here, we report two fast (IgM and IgG), quantitative, and streamlined polarization immunoassays to diagnose LB (FPIA-LB) in a few minutes using genus-specific synthetic peptides. This study also suggests that the FPIA-LB can rapidly manage patients within an emergency department due to its improved sensitivity and specificity, as well as reduced time for assay analysis.

## 2. Results

### 2.1. Epitope Selection

A complete SPOT-synthesis analysis identified 13 linear B-cell IgM [52] and 16 IgG epitopes [18] for *Borrelia burgdorferi* that were recognized by patients’ sera in the five outer membrane proteins Fl 41kD, flagellar hook-associated protein, flagellar hook k2 protein, putative Omp BURGA03, and 31 kDa OspA lipoprotein] from *B. burgdorferi.* The primary goal of these works was to identify antigenic determinants that could differentiate LB infections from closely related pathogens, so bioinformatics was used to BLAST v 2.17.0 the sequences. Two epitopes that originated from proteins Fl 41kD (FITCpep1M) and Flg Hook 2 protein (FITCpep2G) were determined to meet the criteria for a high potential to avoid cross-reactivity based on the absence of multiple sequential amino acids that were identical to segments in other pathogens, including the most highly similar bacteria. The FITCpep1MG and FITCpep2G are recognized specifically by antibodies IgM and IgG, respectively, and were chosen for further analysis.

### 2.2. Peptide Titration

**Figure 1:**
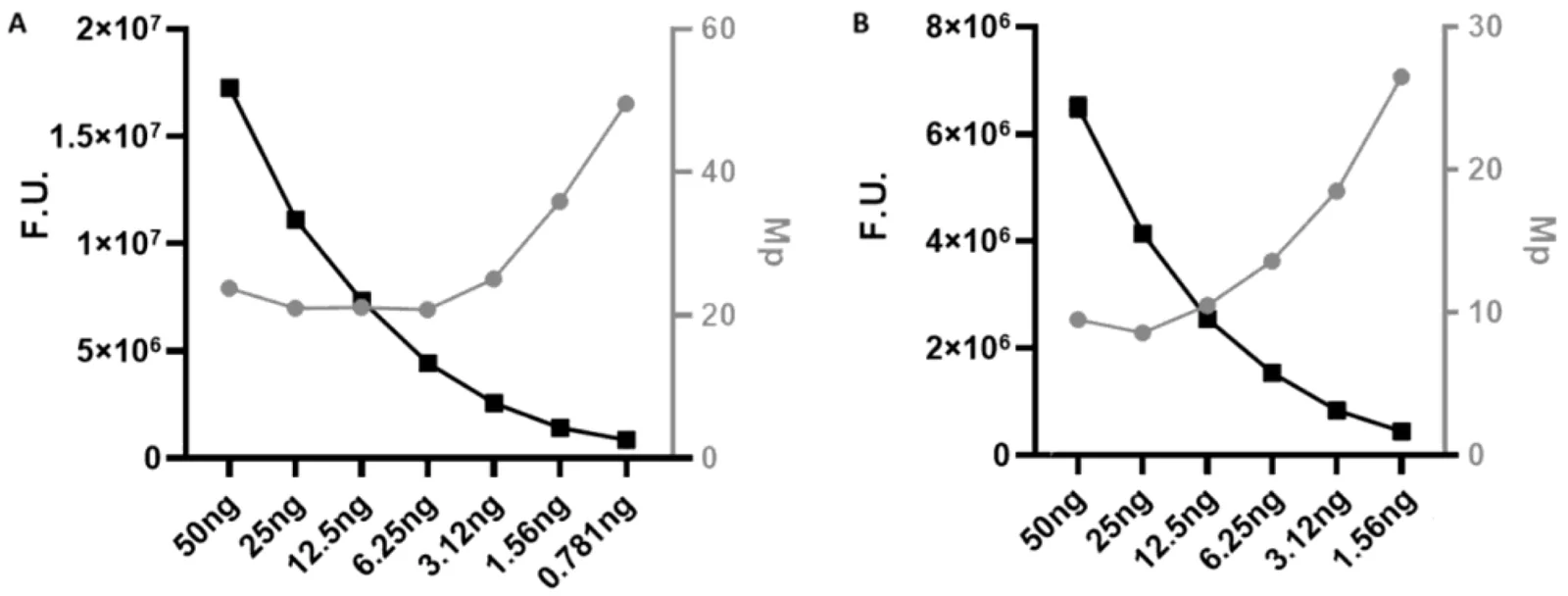
Correlation curves between fluorescence (black squares) and fluorescent polarization (grey circles) for the peptide recognized by IgM antibodies (FITCpep 1M; (**A**)) and IgG antibodies (FITCpep2G, (**B**)). Arrows indicate the concentrations used for each test. F.U., fluorescence Units; Mp, Millipolarization. The results represent the mean of three experiments.

Based on the results obtained with the fluorescent peptides FITCpep1 and FITCpep2, a correlation was observed between the decrease in fluorescence intensity and the increase in fluorescent polarization. During the formation of the titration curve, the intensity of the fluorescence emitted by the peptides progressively decreases with the decrease in concentration in the solution. On the other hand, the fluorescent polarization values demonstrate an inverse trend, with a progressive increase in fluorescent polarization as the concentration of the peptides decreases, a behavior associated with greater rotational freedom of the peptides in solution. Figure 1 shows the correlation analysis between the fluorescence (black squares) and fluorescent polarization (grey circles) for the peptides FITCpep1M recognized by antibodies IgM and FITCpep2G for peptide IgG in solution with their particularities, which were used to select the best test conditions.

### 2.3. Evaluation of Pool Reactivity

**Figure 2:**
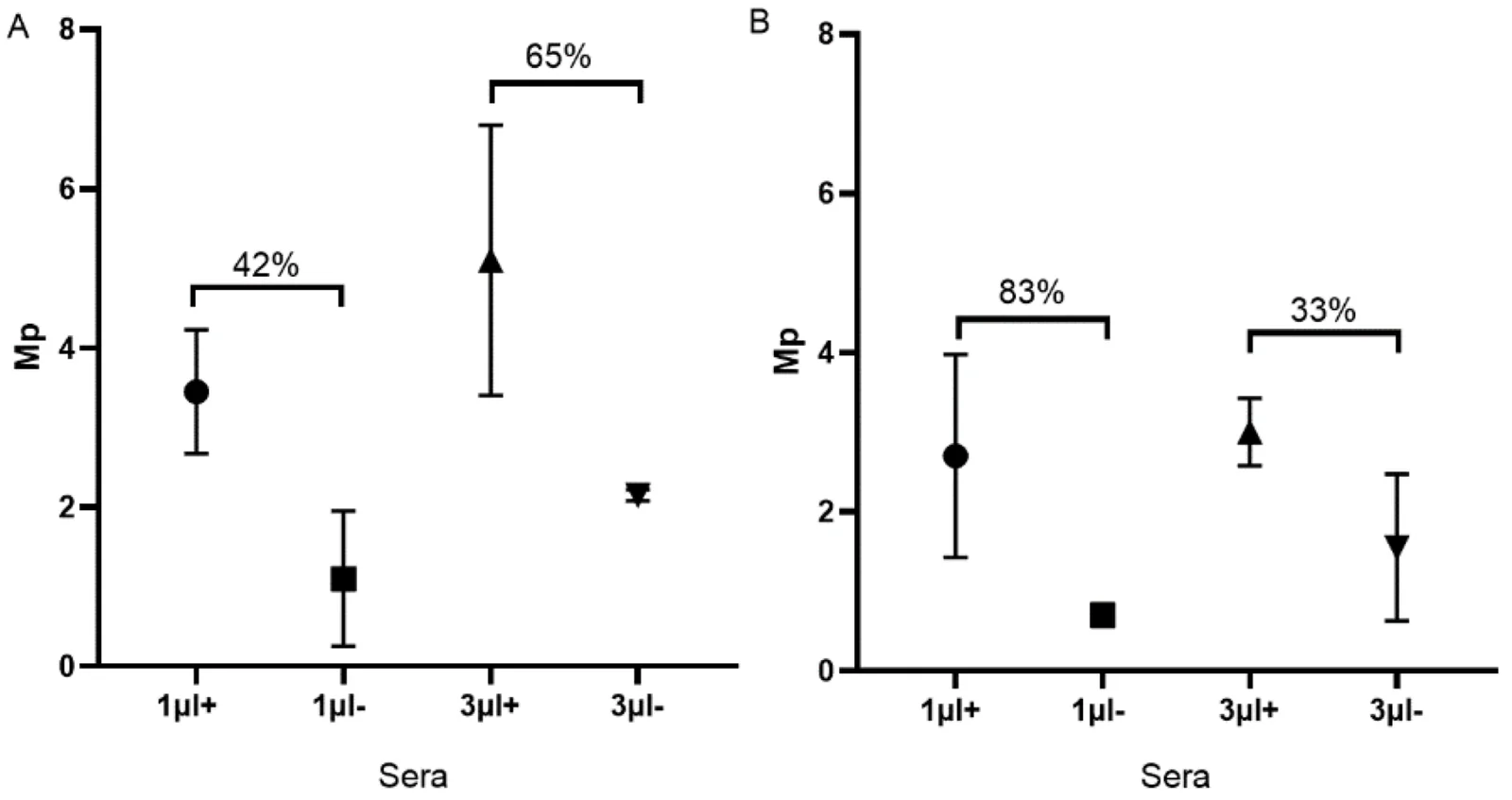
Reactivity of the peptide FITCpep1M (25 ng) with different concentrations of a pool (n = 10) of sera from LB-infected (+) patients and healthy (−) individuals, with reaction times of 5 min (**A**), 30 min (**B**), and temperature of 26 °C. Mp, Millipolarization. The experiment represents the media of duplicate.

During the tests performed with higher concentrations of FITCpep1M, an instability related to the reaction time of 5 and 30 min was noticed due to the differences in the Millipolarization values between the concentrations of 1 µL and 3 µL of the serum pool (Figure 2). The tests continued with a lower peptide concentration (3.12 ng) to evaluate another variable.

**Figure 3:**
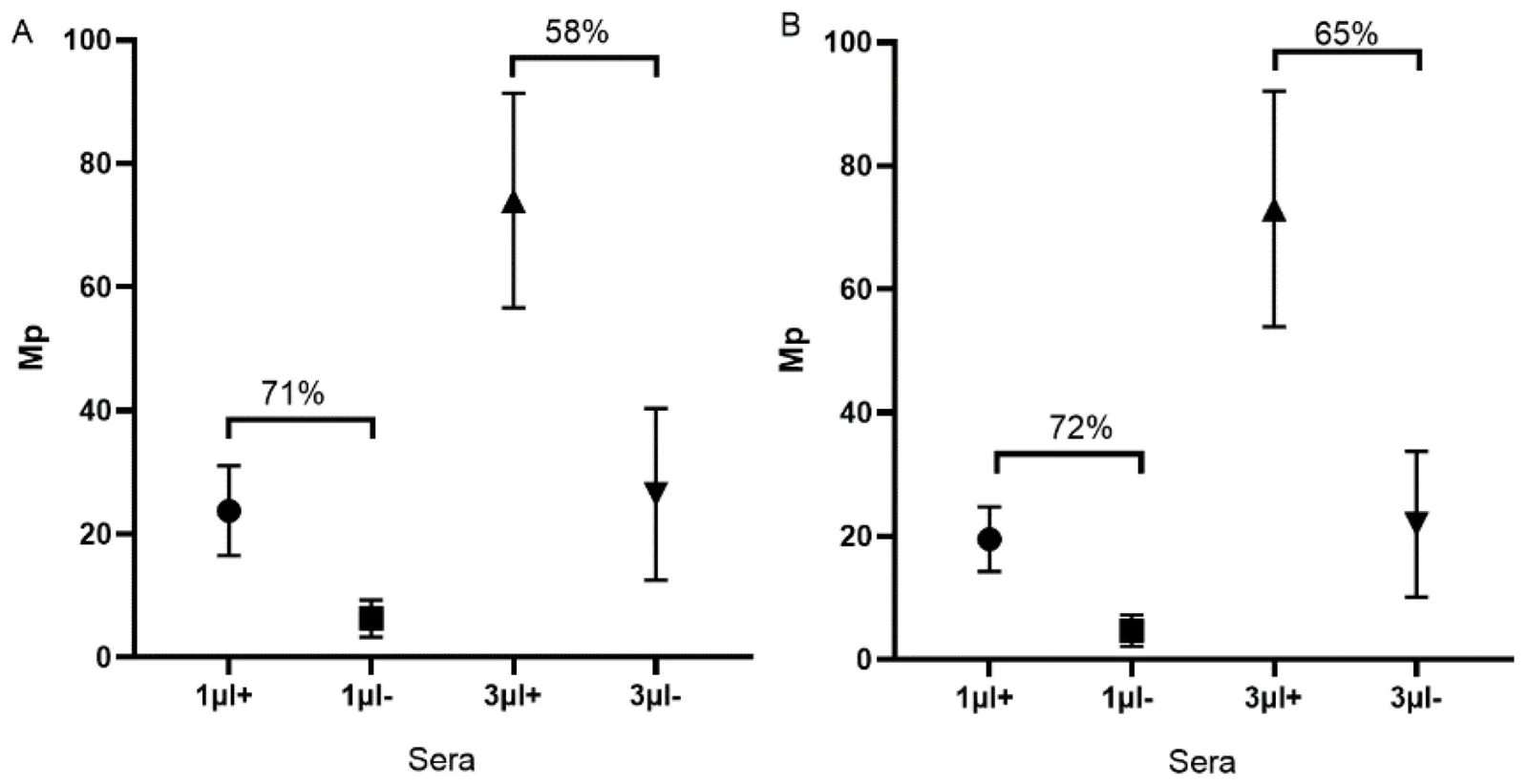
Reactivity of the peptide FITCpep1M (3.12 ng) with different concentrations of a pool (n = 10) of sera from LB-infected (+) patients and healthy (−) individuals, with reaction times of 5 min (**A**) and 30 min (**B**) and temperature of 26 °C. Mp, Millipolarization. The experiment represents the media of duplicate.

**Figure 4:**
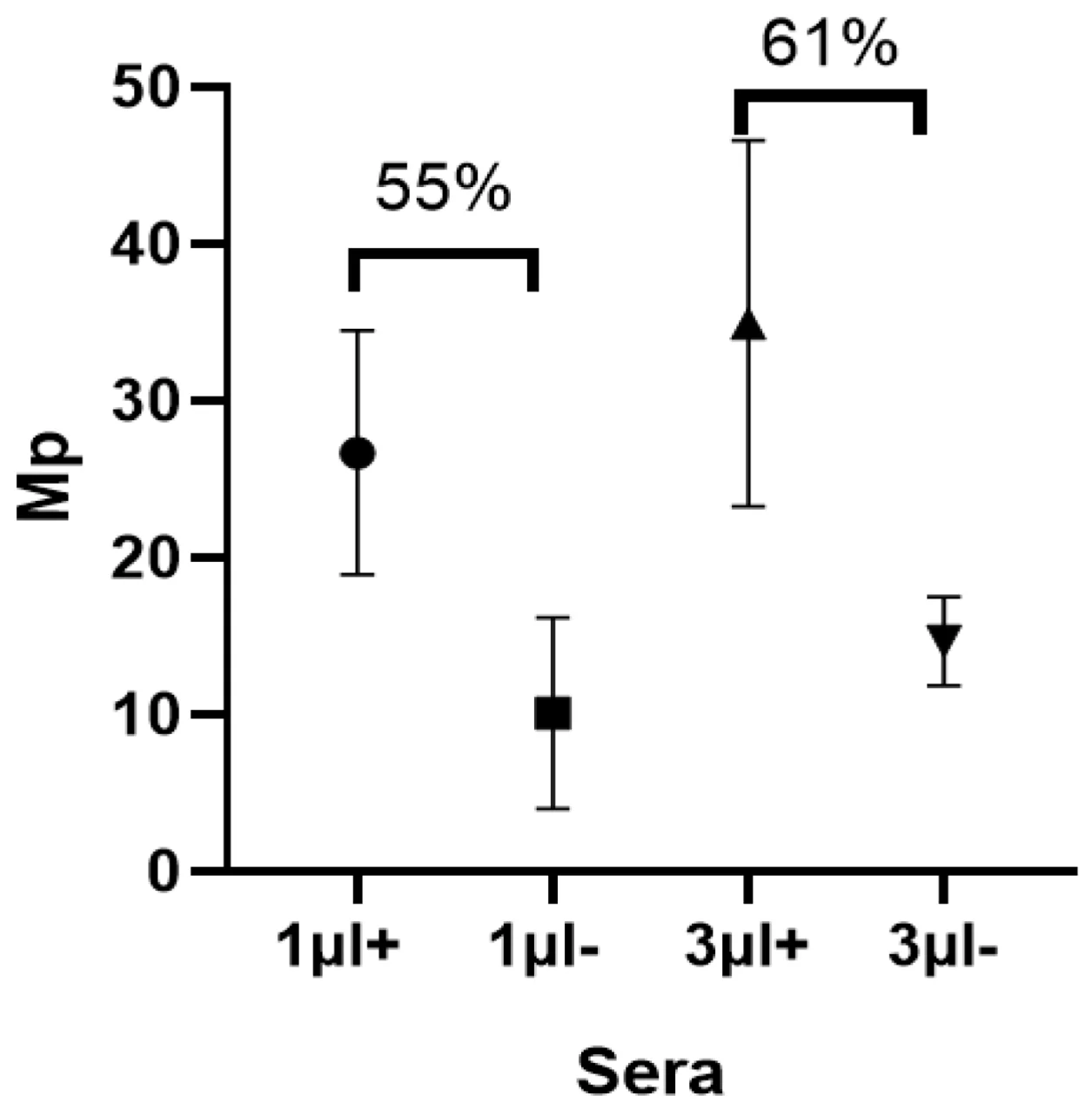
Reactivity of the peptide FITCpep2G (3.12 ng) with different concentrations of a pool (n = 10) of LB-infected (+) patients’ sera and healthy (−) individuals, with reaction times of 5 min at 26 °C. Mp, Millipolarization. The experiment represents the media of duplicate.

By reducing the concentration of FITCpep1M (Figure 3), greater stability was noticeable between the reaction times, which remained stable. For FITCpep2G, the selected peptide concentration was the same as that of the test with the already standardized FITCpep1M (3.12 ng), using a 5-min reaction time (Figure 4), but with a larger quantity of the serum pool selected, as it presented greater reactivity.

**Figure 5:**
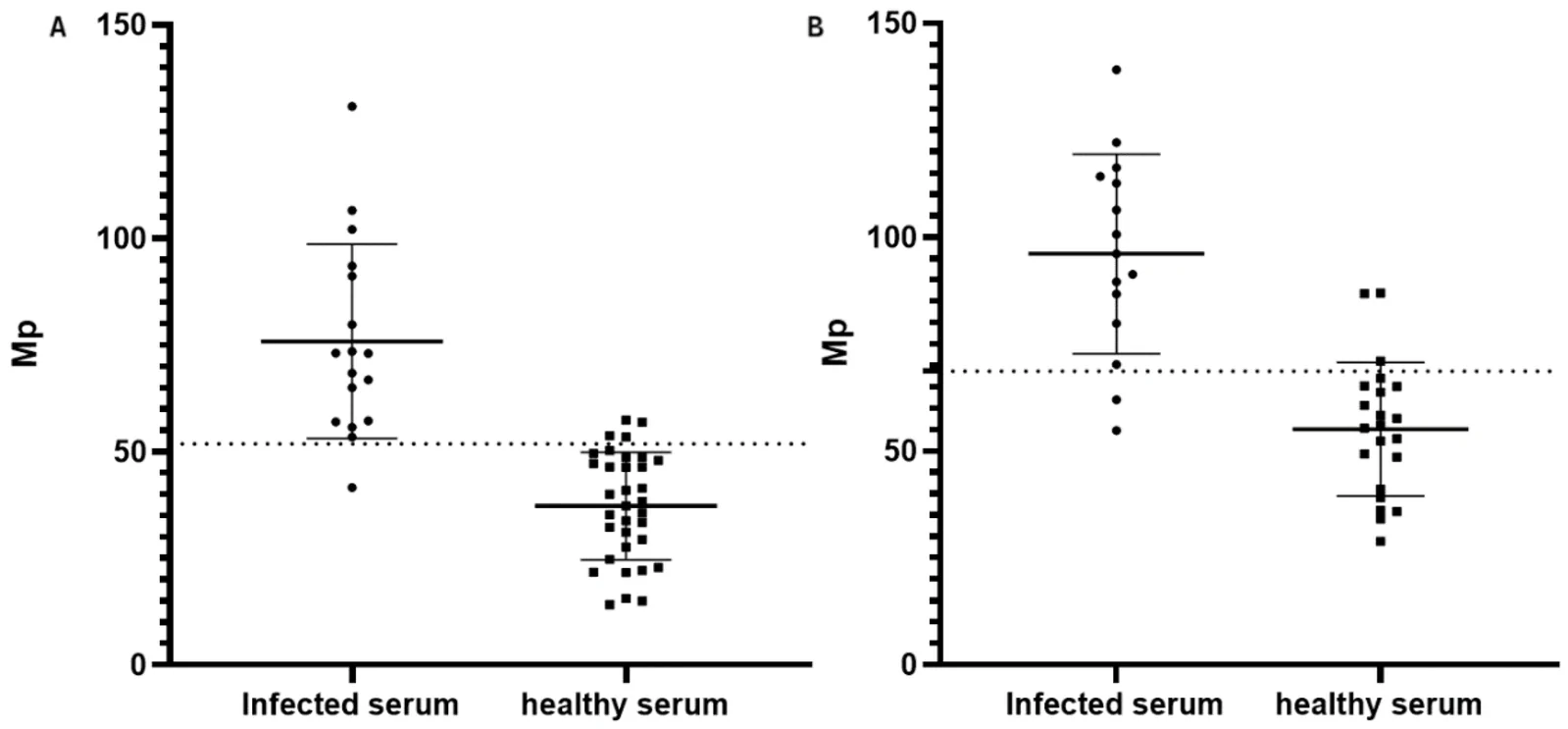
FPIA-LB assay showing the reactivity of FITCpep1M ((**A**); 3.12 ng) and FITCpep2G ((**B**); 3.12 ng) with LB patients’ sera (n = 19) and healthy individuals (n = 38) in 5 min of reaction at 26 °C. Mp, Millipolarization.

The results of the fluorescent polarization assay with FITCpep1M using individual sera were promising for separating infected and healthy sera. It was possible to observe a separation in the mean polarization (mP) values (Figure 5), with the positive sera presenting high values, using 19 positive and 38 negative sera. In contrast, the negative samples maintained low values for FITCpep2G; 15 infected and 23 healthy sera were tested, also yielding a high separation in the values between the two.

**Figure 6:**
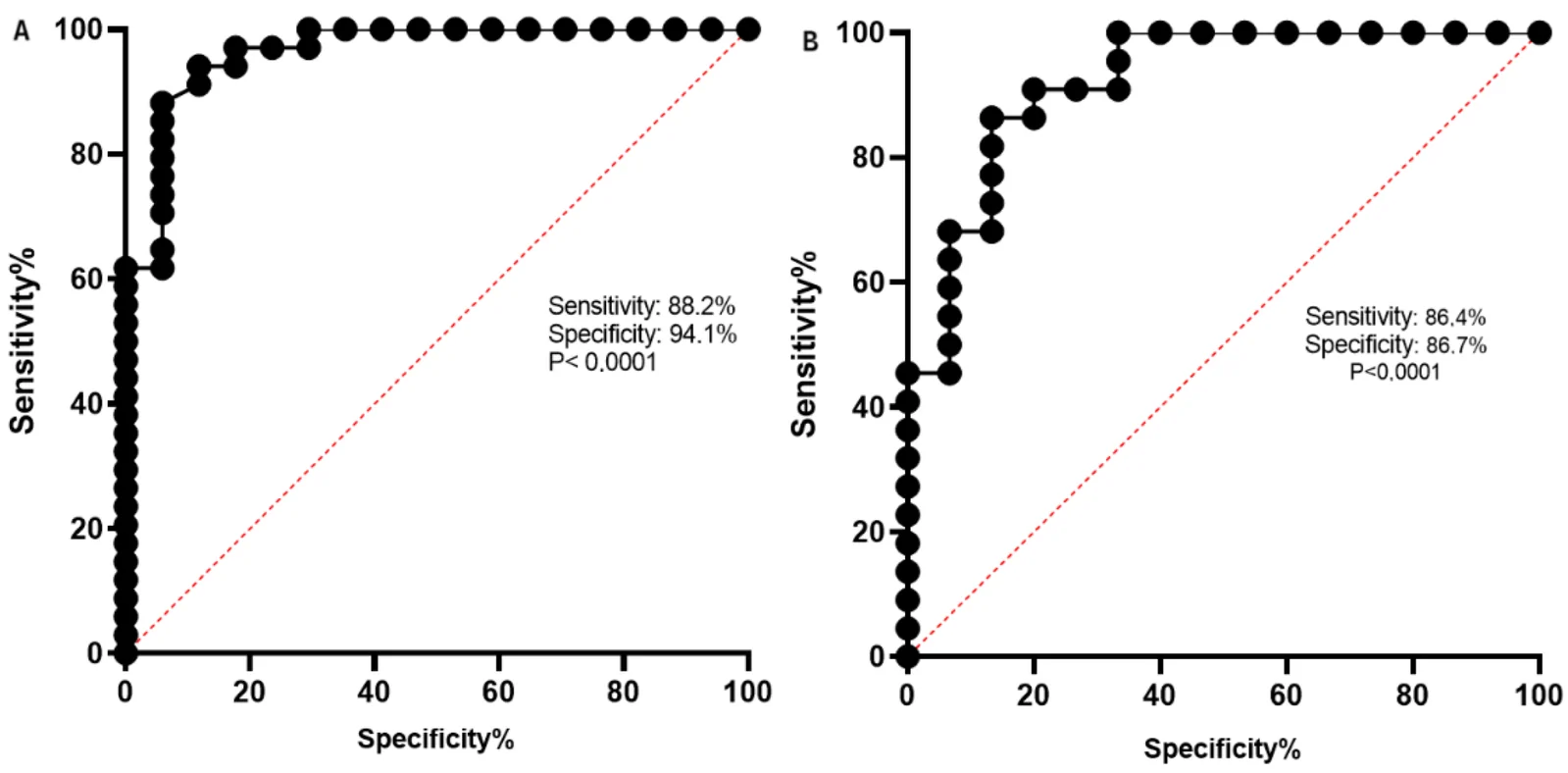
The ROC curve shows the trade-off between sensitivity and specificity for the FITCpep1M (**A**) and FITCpep2G (**B**), with *p* value < 0.0001. A sensitivity of 88.2% and specificity of 94.1% was obtained for the FITCpep1 (**A**), and a sensitivity of 86.4% and specificity of 86.7% for the FITCpep2 (**B**).

The evaluation of the ROC curve (Figure 6) confirmed the efficacy of the technique, showing a sensitivity of 88.2% and a specificity of 94.1% for FITCpep1 and 86.4% sensitivity and 86.7% specificity for FICTpep2, indicating that the experiment can adequately identify most positive cases (low false-negative rate) and adequately recognize negative cases (low false-positive rate), with a *p* value of < 0.0001 for both experiments.

## 3. Discussion

Accurate diagnosis of LB and early detection are crucial to preventing chronic complications, such as Lyme arthritis, carditis, and neuroborreliosis. Conventional diagnostic methods involve a two-tiered testing algorithm: first, antibodies against Borrelia are identified through ELISA, followed by Western blot, a protocol recommended by the CDC (Centers for Disease Control and Prevention). Although these methods exhibit good sensitivity, they have limitations, including delays in obtaining results and low specificity [53,54]. However, the potential for high-throughput testing holds promise for Lyme disease diagnostics, offering faster and more accurate results and instilling hope in the medical community.

The reactivity of human IgM with the outer surface protein C (OspC) of *Borrelia burgdorferi* sensu lato is frequently used to detect *Borrelia-specific* IgM in commercial immunoassays, and such antibodies usually occur in the early phase of the infection [55]. This procedure identifies IgM antibodies directed against *Borrelia* antigens in serum samples, demonstrating high sensitivity. However, the specificity of this and other assays using different proteins may be compromised by cross-reactivity with other spirochetal infections, such as *Leptospira integrans* and *Treponema pallidum* [56], and viral infections, such as Epstein-Baar virus and Cytomegalovirus [57,58,59], and IgG anti-*Borrelia* antibodies in *Yersinia* which increases the likelihood of false-positive results [54]. Western blotting, performed in sequence, examines the reactivity of antibodies against specific proteins of Borrelia. This procedure improves accuracy by identifying typical band patterns. Despite being more complex, it involves cross-reactivity [59], necessitating manual methods and subjective interpretation, which can lead to laboratory variations [60]. These drawbacks can lead to misdiagnoses or delayed treatments, particularly in cases where prompt clinical decisions are crucial, underscoring the urgency of the issue.

Molecular diagnostics, such as polymerase chain reaction (PCR), offer enhanced specificity and sensitivity for identifying *Borrelia* DNA. However, these techniques are generally restricted to centralized laboratory settings, making them less accessible for immediate clinical use. Therefore, this highlights the urgent need for point-of-care diagnostic tools that deliver accurate, rapid results directly to patients.

As an alternative, fluorescent polarization emerges as an innovative, rapid, and accurate approach to diagnosing Lyme disease. This technique is based on the difference in the degree of rotation of a fluorophore bound to the free peptide or when it interacts with a larger molecule, such as an antibody or target protein, generating different fluorescent polarization signals. The main advantage of this technique is its ability to directly measure molecular interactions without requiring washing steps, thereby reducing analysis time and minimizing technical errors [61].

The behavior observed in both peptides, where fluorescence values decrease as the peptide-fluorophore is diluted and polarization values increase inversely, demonstrates the relationship between the concentration of the peptide-fluorophore and its rotation. Serial dilution decreases the amount of peptide-fluorophore in the solution, reducing the density of excitable molecules. Thus, the intensity of the emitted fluorescence also decreases in proportion to the concentration. This relationship is common in fluorescence experiments if there are no major external interferences, such as quenching of the fluorophores [62].

Molecular rotation during the emission time is inversely proportional to fluorescent polarization [63]. At high concentrations, interactions between molecules, such as aggregation, can restrict rotational motion, decreasing FP values. At lower concentrations, these interactions are reduced, and polarization increases since the molecules have greater freedom of rotation [41].

High concentrations of peptide-fluorophores can result in undesirable consequences, such as aggregation, impairing the system’s stability by decreasing the linearity of the response. Therefore, lower concentrations are preferable since they reduce these effects and ensure greater reliability of the results [63].

Two initial concentrations of FITCpep1M were tested, but only one concentration of FITCpep2G was chosen, as it closely matched the accurate test for the first peptide. The choice of concentration closest to 3.12 ng was based on technical criteria that considered establishing a concentration that would guarantee a robust fluorescent signal with a high signal-to-noise ratio while minimizing potential interference and receptor saturation in the system. This ensures that the chosen concentration is optimal for the test.

The reaction data observed in Figure 1, with 25 ng of FITCpep1M and a pool of sera, demonstrate significant instability in the millipolarization values between the 5- and 30-min reaction times. In contrast, the same analysis performed with 3.12 ng of peptide presented stable curves throughout the entire time. The probable explanation is that high concentrations of the peptide can lead to an imbalance in the interaction with the analyte since the time required to reach dynamic equilibrium increases. These slower kinetics may be responsible for the variations observed between the times.

When using 3.12 ng of FITCpep1M, the large variation in reactivity between the times was not observed. For this reason, the 5-min time was selected, as it can be used as a favorable factor in developing a test with a shorter processing time. In addition, it was possible to observe that a smaller amount of patient serum resulted in a greater difference between infected and healthy samples, and therefore, it was selected.

For FITCpep2G, the 5-min time was maintained, with a concentration of 3.12 ng. However, a higher serum concentration from the pool was necessary to increase the difference between infected and healthy individuals, possibly due to the difference in the concentration of IgG compared to IgM in the patient’s serum. For this, 3 µL was chosen for the tests and performed well.

The results for the two peptides analyzed in the fluorescent polarization test with individual sera showed different diagnostic performances. However, both could differentiate between infected and healthy individuals. FITCpep1M demonstrated a sensitivity of 88.2% and specificity of 94.1%.

The superior efficacy of FITCpep1M, with a sensitivity of 88.2% and a specificity of 94.1%, indicates its greater affinity and specificity with the antibodies present in the sera of infected patients. This leads to an increase in the identification of true-positive cases and a decrease in the occurrence of false positives. In comparison, FITCpep2G exhibited a sensitivity of 86.4% and a specificity of 86.7%. These results align with the literature, which indicates that this immunoglobulin is crucial in the early diagnosis of Borreliosis [64] and persists longer in the blood of infected patients with and without symptoms [55], a finding that may suggest active infection.

As antibodies are versatile tools in the immune system’s toolkit, each isotype is designed to tackle specific challenges. Each isotype may prefer certain epitopes or amino acid residues, which may be better suited to deal with particular types of invaders. Therefore, the behavior of the antigenic epitopes analyzed in this study may be linked not only to the concentration of IgM but also to the structural variability of specific IgM (pentameric and hexameric) [65], which may favor better interactions with the antigen FITCpep1M.

In contrast, FITCpep2G, despite exhibiting slightly lower sensitivity and specificity values, still exhibited good diagnostic performance. Although this peptide exhibited lower sensitivity and specificity, the reduced sensitivity may be linked to efforts aimed at improving specificity [66,67]. By increasing the specificity of the test (reducing false positives), sensitivity may be compromised, leading to a higher rate of false negatives. This balance between sensitivity and specificity is a common challenge in diagnostic testing, where attempts to improve one measure can negatively affect the other.

The ROC curves of both peptides highlight their ability to distinguish between positive and negative sera, indicating the superiority of FITCpep1M in striking a balance between sensitivity and precision. Complementary research involving a larger number of samples and varied experimental conditions may enhance the analysis of each peptide and allow for modifications to improve the total yield, such as small changes in sequence or incubation conditions.

These tests highlight the effectiveness of the fluorescent polarization technique in diagnosis and emphasize the importance of carefully choosing the peptide used, considering both diagnostic efficacy and the practical aspects of its clinical use.

A comparison of FPIA and ELISA methods has demonstrated that both methods have similar sensitivity and specificity. While both techniques may offer comparable sensitivity, FPIA generally requires a smaller sample volume and provides faster analysis times compared to ELISA, which often involves multiple steps and longer processing periods. Additionally, FPIA’s ability to provide rapid, real-time results could be particularly advantageous in point-of-care settings, offering timely diagnosis and treatment decisions [30,68,69]. However, FPIA has a shorter analysis time and is easier to perform. The undoubted advantage of FPIA over ELISA is the lower analysis cost, as this format requires only one reagent, a fluorescently labeled antigen, and a shorter test completion time.

Furthermore, based on the results obtained, the technique can be implemented in high-throughput formats, such as 384-well plates, facilitating the simultaneous analysis of multiple samples with low volume. In the future, fluorescent polarization assays may become a more efficient alternative to traditional methods, especially in mass screening scenarios where rapid diagnosis is crucial. Their integration with automated and miniaturized platforms potentially further expands their accessibility and impact in diagnosing Lyme borreliosis and other infectious diseases.

## 4. Materials and Methods

### 4.1. Human Serum Samples and Ethical Considerations

Human serum samples were obtained from the Rheumatology Division at the University of São Paulo (USP/São Paulo), School of Medicine. The panel comprised 19 samples of seropositive patients clinically diagnosed with LB-like syndrome and confirmed by PCR, Western blot, and ELISA tests. Additionally, the study included 38 seronegative samples from a healthy blood center, HEMORIO (Arthur de Siqueira Cavalcanti State Institute of Hematology, Rio de Janeiro, Brazil). The Ethics in Research Committee of FIOCRUZ (CEP-25836019.0.0000.5243) and USP (CAAE 25836019000005243) approved the experiments involving human serum samples, which were conducted by good clinical practice and all applicable regulatory requirements, including the Declaration of Helsinki.

### 4.2. Epitope Mapping and Selection of Peptide Sequences

The complete sequences of five outer membrane proteins [Fl 41kD (PI1089), flagellar hook-associated protein (Q44767), flagellar hook k2 protein (O51173), putative Omp BURGA03 (Q44849), and 31 kDa OspA (P0CL66)] lipoproteins from *B. burgdorferi* were immunologically mapped by microarrays of peptides based on the Spot-synthesis technology as described previously [18]. Fourteen IgM and nineteen linear IgG epitopes were identified using patient sera infected with Borrelia, and two specific epitopes without reaction with syphilis and leptospirosis patients’ sera were selected for use in this study. The IgM epitope PGLESKYN (Bburg/06/huM, derived from Flg41 kDa) [50] and the IgG epitope [12] SNEDQPNNY (Bburg/12/huG, derived from Flg Hook 2) (here renamed as FITCpep1M and FITCpep2G, respectively) were selected for this study.

### 4.3. Solid Phase Peptide Synthesis

Peptides (PGLESKYN (pep1M)) and (SNEDQPNNY (pep2G)) were synthesized using the F-moc strategy on a synthesizer machine (MultiPep-1 CEM Corp, Charlotte, NC, USA) as described previously [70]. After sequence assembly, a fluorescein molecule (FITC) was added at the N-terminal region, the F-moc groups were removed, and the peptide resin (NovaTag^®^, Sigma-Merck, St Louis, MO, USA) was cleaved and completely deprotected with TFA/H2O/EDT/TIS (94/2.5/2.5/1.0 *v*/*v*, 90 min). The peptides (FITC-pep1M and FITC-pep2G) were precipitated with chilled diethyl ether and centrifuged, and the pellet was reconstituted in aqueous AcOH (10% *v*/*v*), dried, and stored as lyophilized powder. Peptides, when required, were dissolved in water and then centrifuged; the resulting supernatant was subsequently filtered using a Centricon-10 filter. Single peptides were used without prior purification, with their identity confirmed by MS (MALDI-TOF-MS MicroflexLT instrument; Bruker Daltonics, Bremen, Germany).

The Nanodrop-1000 spectrophotometer (Thermo-Fisher Scientific, Waltham, MA, USA) v3.8 quantified the peptides using a 2.5 µL sample from a 1 mL stock solution. To accurately quantify the peptide sequences, molecular weights were determined using the ProtParam program [71], and the following formula was applied: (A.U. × M.W.)/Extinction Coefficient (https://web.expasy.org/protparam/; accessed on 10 March 2022).

### 4.4. Fluorescent Polarization Immunoassay

Fluorescent polarization was measured in a Hidex spectrophotometer plate reader (Hydex Oy, Turku, Finland) using 480 nm (ex) and 520 nm (em). The FITCpep1M and FITCpep2G peptides were diluted in TBST (Tris-buffer saline plus 0.05% Tween-20), and 50 µL of the solution was added to a Nunc 96-Well black plate (Thermo-Fisher Scientific, Waltham, MA, USA) with a transparent bottom. The curves were measured in triplicate at a temperature of 26 °C. One microliter and 3 µL from the pool (n = 10) of sera from LB-infected patients and healthy individuals were used to evaluate the reactivity of the antibodies in 5- and 30-min reaction times. The individual evaluation followed the values stipulated in the standardization.

### 4.5. Statistical Analysis

The data relating to the raw values obtained by the Hidex spectrophotometer were transformed into figures using the GraphPad-Prism and Excel programs (GraphPad Software, Inc., San Diego, CA, USA). These programs also calculated cut-off values for the receiver operating characteristic (ROC) and *p* values.

## 5. Conclusions

The FPIAs, developed around a unique synthetic peptide, offer a significant advancement in the field of diagnostics. They provide a one-step solution for detecting IgM and IgG antibodies against LB, with a clear distinction between infected and healthy individuals. Their sensitivity and specificity are comparable to those of ELISAs, but they surpass them with their rapid results, the absence of secondary antibodies, and simplified procedural steps. Their streamlined workflow also presents opportunities for scalability, making them a valuable addition to clinical applications.

Rapid, point-of-care solutions enhance diagnostic accuracy, empowering healthcare providers to initiate timely and targeted treatments that ultimately improve patient outcomes and reduce the burden of this complex disease. In this context, the developed FPIAs for detecting the IgM (FITCpep1M) and IgG (FITCpep2G) specific immune responses suspected of having LB represent an advance in creating specific and sensitive serological tests. They require reduced sample volumes and are cost-effective, making them accessible to a broader population, which further reinforces their clinical utility.

The successful development and validation of fluorescence polarization immunoassays (FPIAs) for the diagnosis of Lyme borreliosis highlights the technique’s potential for broader applications in the diagnosis of infectious and non-infectious diseases. Given its high sensitivity, specificity, and rapid results, FPIA can be adapted for the detection of other pathogens. This method could revolutionize the diagnosis of viral infections such as dengue and COVID-19, as well as bacterial diseases, utilizing pathogen-specific synthetic peptides to minimize cross-reactivity and increase diagnostic accuracy.

The technique’s compatibility with high-throughput formats makes it ideal for large-scale epidemiological studies, vaccination campaigns, and public health surveillance, particularly in resource-limited settings. Furthermore, integrating the technique with portable point-of-care devices can decentralize testing, reducing reliance on laboratories and accelerating clinical decision-making.

## Data Availability

Not applicable.
